# Supplementary material for: The relative transmission fitness of multidrug-resistant Mycobacterium tuberculosis in a drug resistance hotspot
Source: Nat Commun. 2023 Apr 8;14:1988. doi: 10.1038/s41467-023-37719-y (PMC10082831; doi:10.1038/s41467-023-37719-y)
Supplement: Supplementary file 1 — Supplementary Information [file 41467_2023_37719_MOESM1_ESM.pdf]

Supplementary Information

The relative transmission fitness of multidrug-resistant *Mycobacterium tuberculosis* in a drug resistance hotspot

Chloé Loiseau, Etthel M. Windels, Sebastian M. Gygli, Levan Jugheli, Nino Maghradze, Daniela Brites, Amanda Ross, Galo Goig, Miriam Reinhard, Sonia Borrell, Andrej Trauner, Anna Dötsch, Rusudan Aspindzelashvili, Rebecca Denes, Klaus Reither, Christian Beisel, Nestani Tukvadze, Zaza Avaliani, Tanja Stadler, Sebastien Gagneux

Table of Contents

1. Supplementary figures 2

2. Supplementary tables 15

1. Supplementary figures

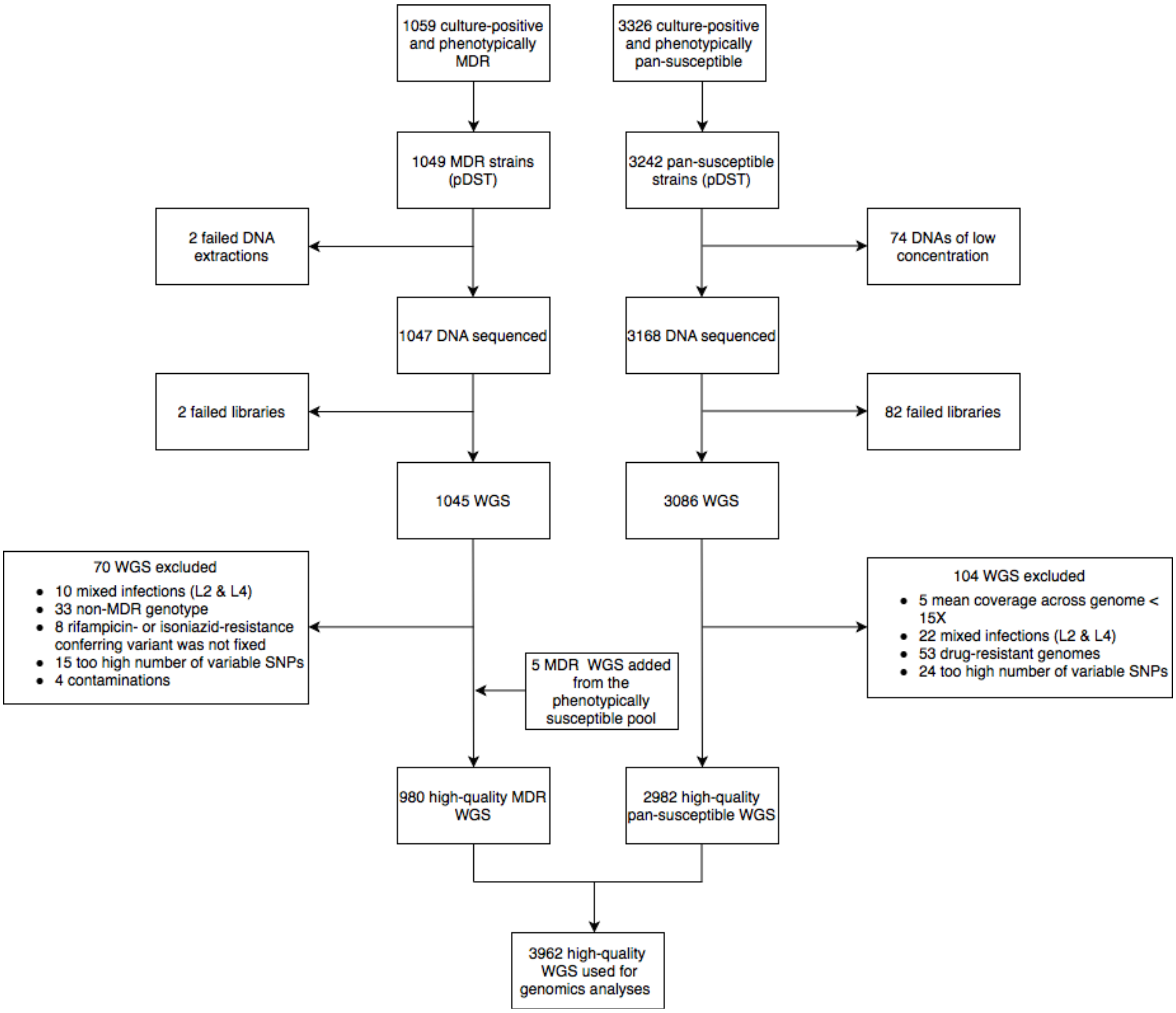

Fig. S1. Flowchart of the data collection strategy.

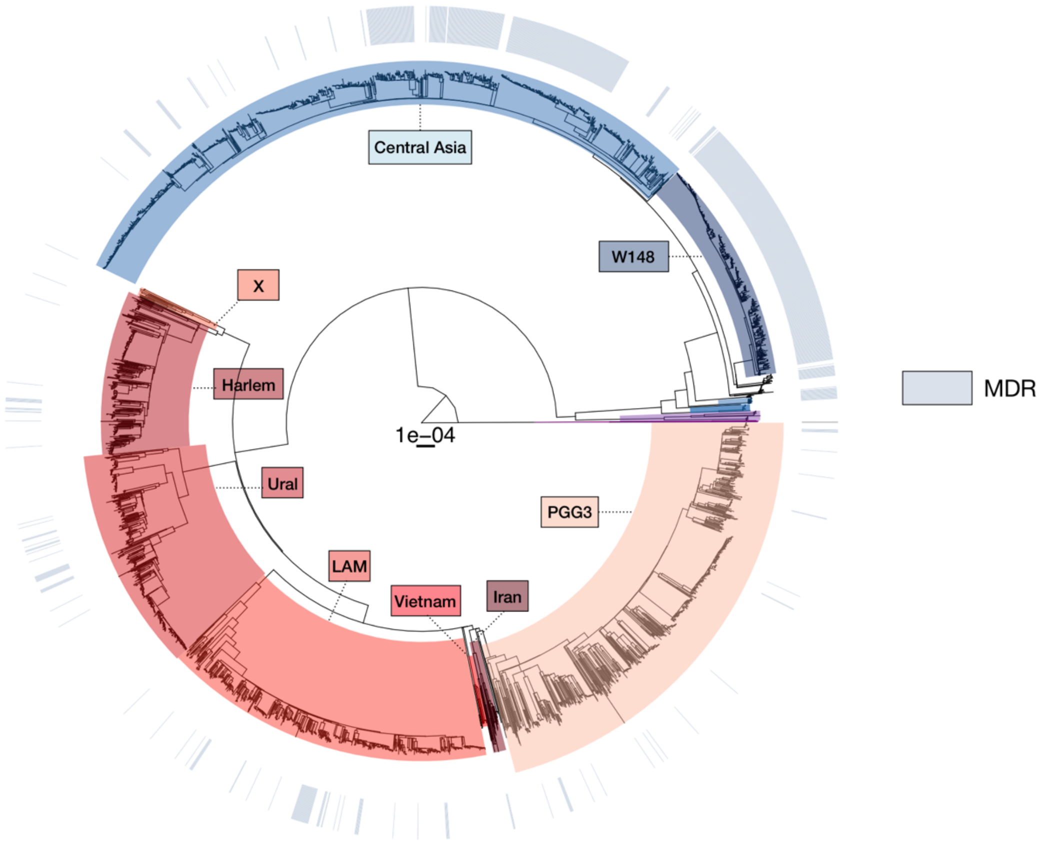

Fig. S2. Maximum likelihood phylogenetic tree of the entire dataset (3,962 genomes), based on 71,219 variable SNP positions and rooted on *Mycobacterium canettii*. Lineage 2 strains are colored in blue with the main sublineages depicted in different shades of blue. Lineage 4 strains are colored in red with the main sublineages depicted in different shades of red. The outer ring corresponds to MDR strains (including MDR strains that carry compensatory mutations). Scale bar indicates substitutions per site.

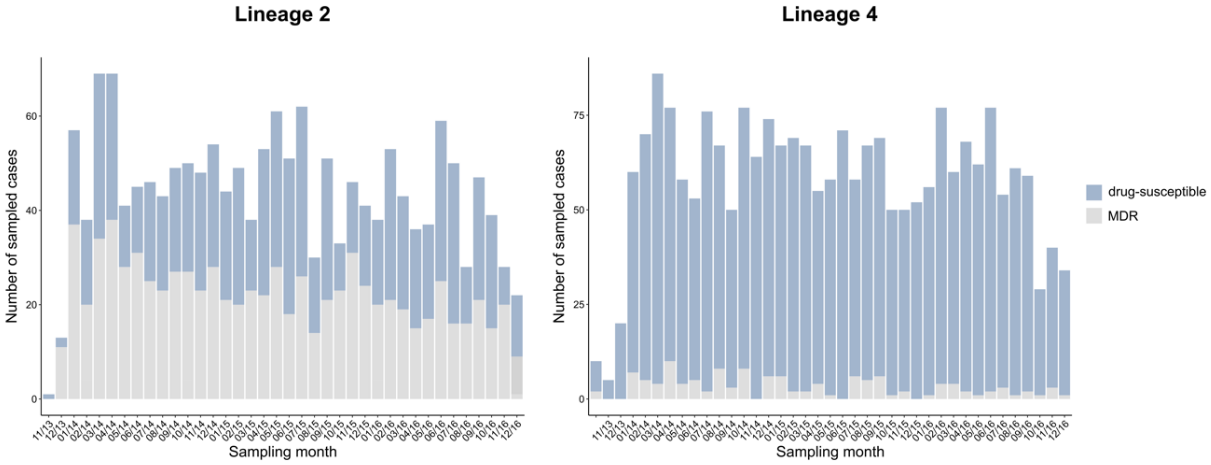

Fig. S3. Prevalence of drug-susceptible and MDR strains over time during the sampling period. Lower case numbers in the first and last month of the sampling period reflect differences in sampling efforts during these months.

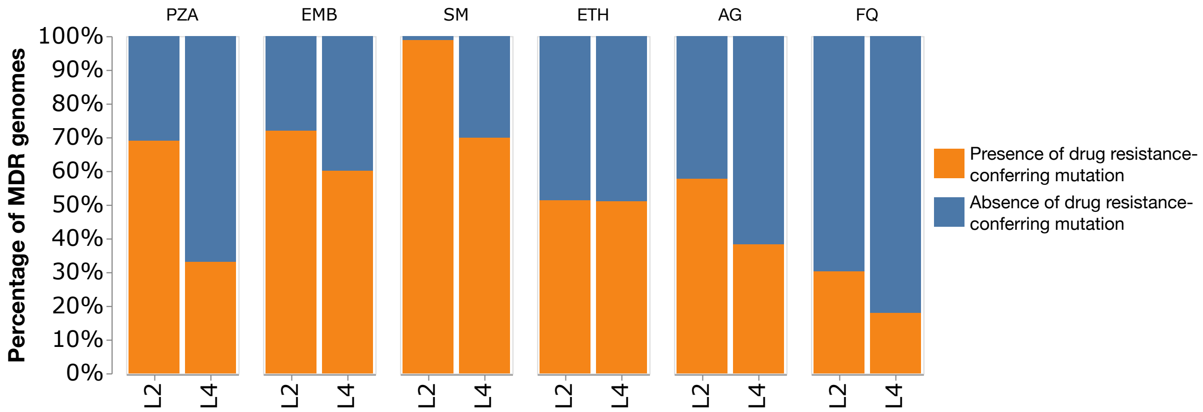

Fig. S4. Proportion of genomes resistant to different drugs in lineage 2 and lineage 4 MDR strains. PZA = pyrazinamide; EMB = ethambutol; SM = streptomycin; ETH = ethionamide; AG = aminoglycosides; FQ = fluoroquinolones.

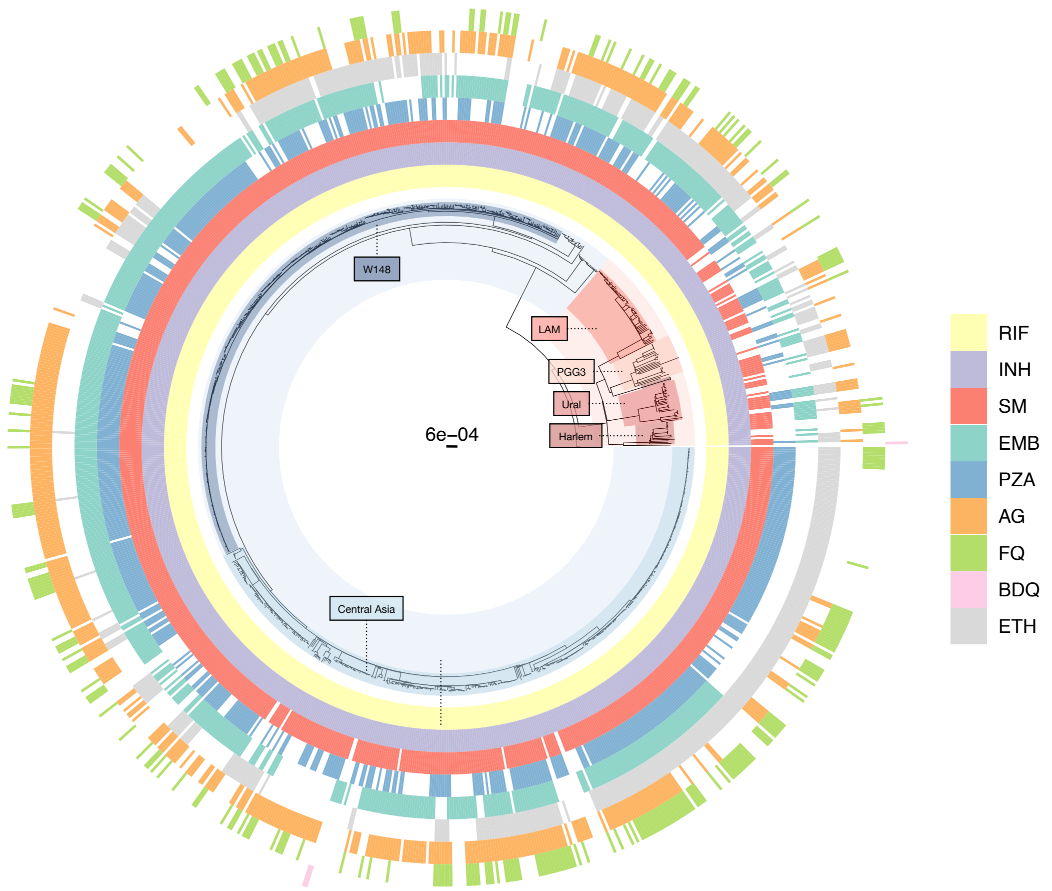

Fig. S5. Maximum likelihood phylogeny of 980 MDR *M. tuberculosis* genomes, constructed from 13,649 variable nucleotide positions. Blue clades indicate lineage 2 strains and red clades indicate lineage 4 strains. Scale bar indicates substitution per site. The outer rings indicate the presence of resistance-conferring mutations to different anti-TB drugs. RIF = rifampicin; INH = isoniazid; SM = streptomycin; EMB = ethambutol; PZA = pyrazinamide; AG = aminoglycosides; FQ = fluoroquinolones ; BDQ = bedaquiline; ETH = ethionamide.

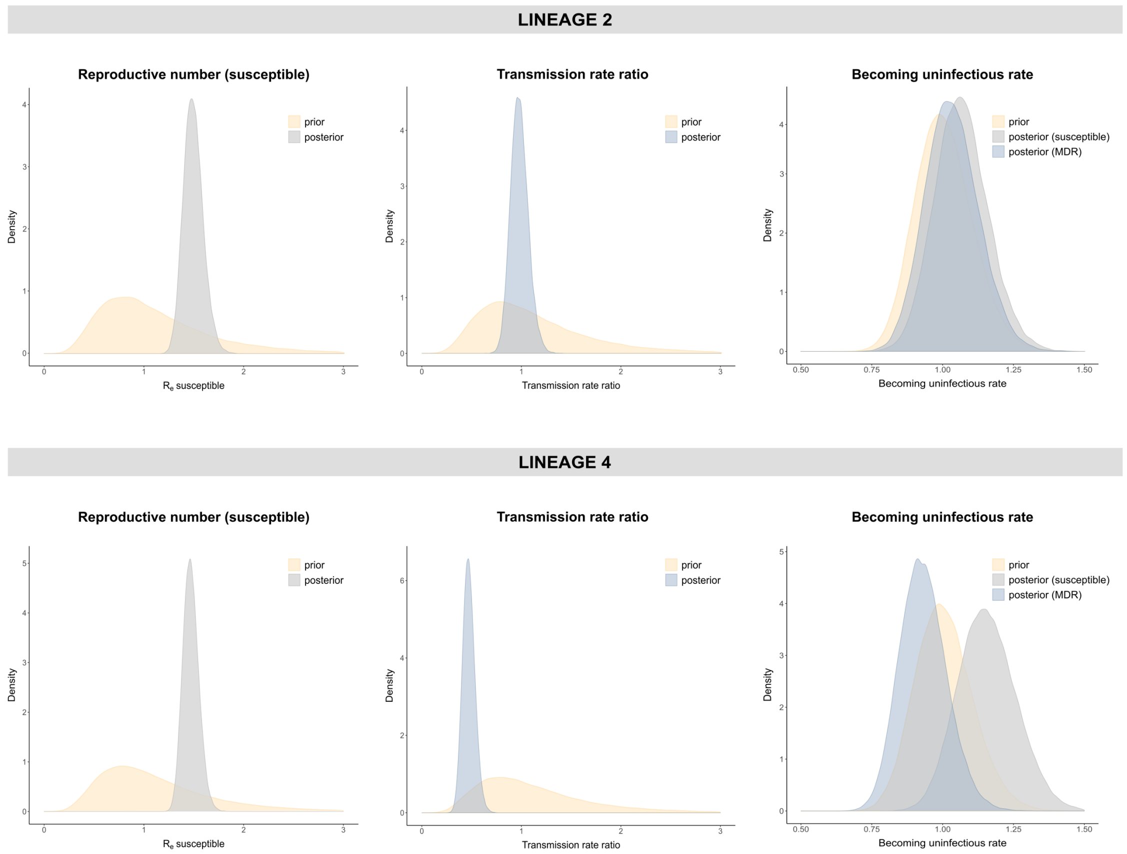

Fig. S6. Prior and posterior distributions corresponding to the phylodynamic analyses shown in Figure 3 of the main text.

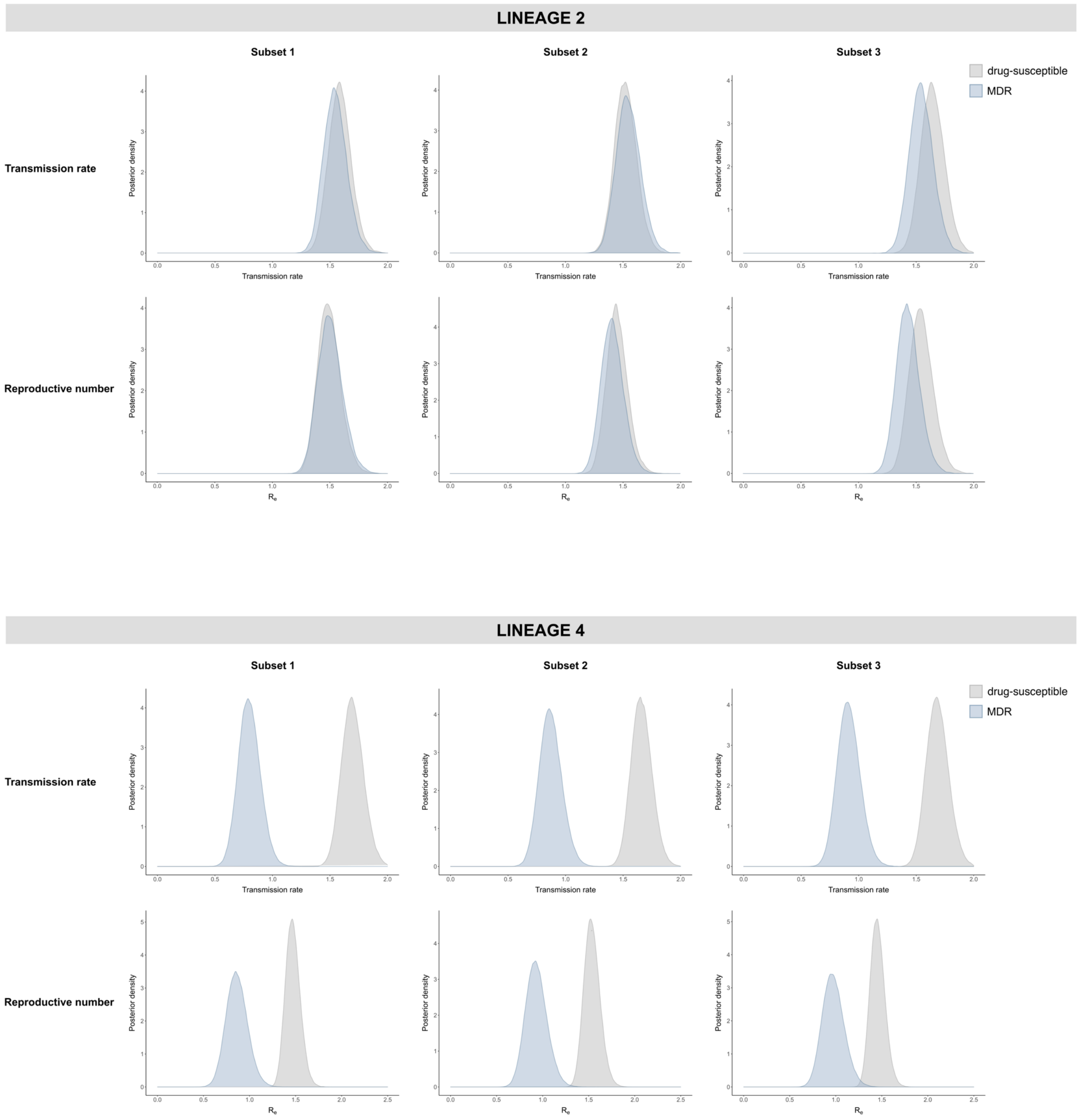

Fig. S7. Posterior distributions resulting from phylodynamic analyses on three random subsets show that the results are robust to subsampling.

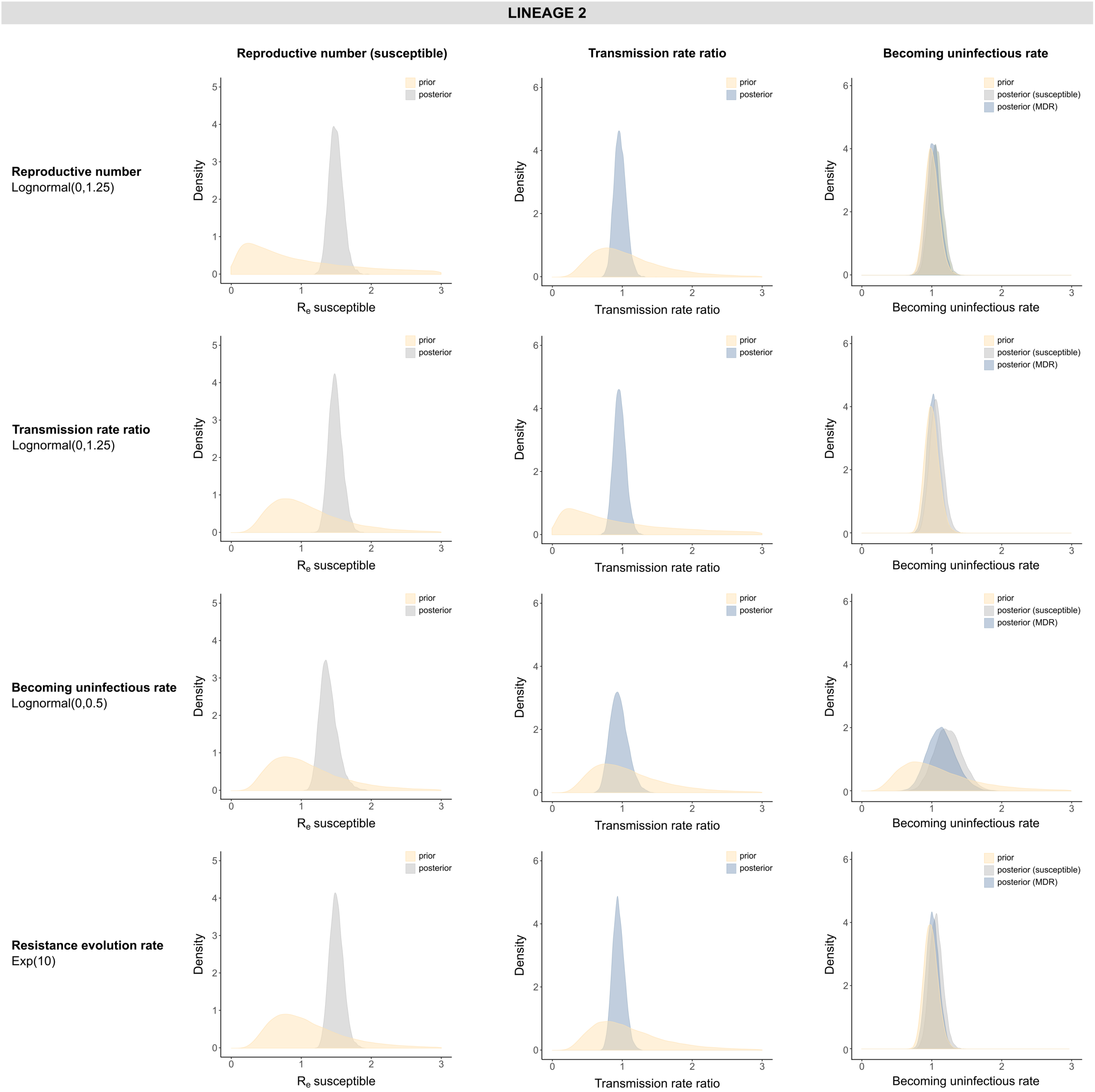

Fig. S8. Results of the phylodynamic analyses are robust to different choices of prior distributions (lineage 2).

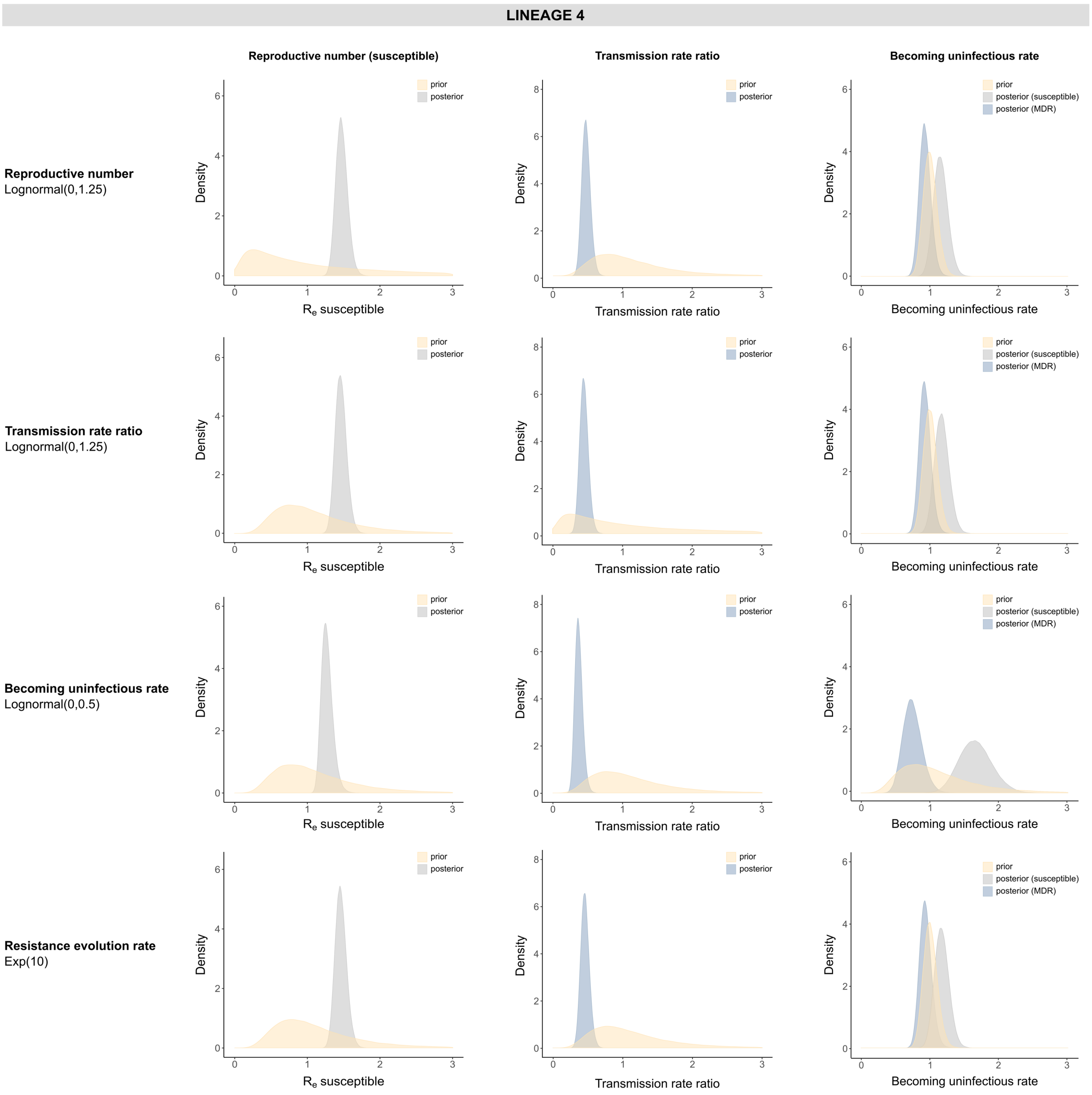

Fig. S9. Results of the phylodynamic analyses are robust to different choices of prior distributions (lineage 4).

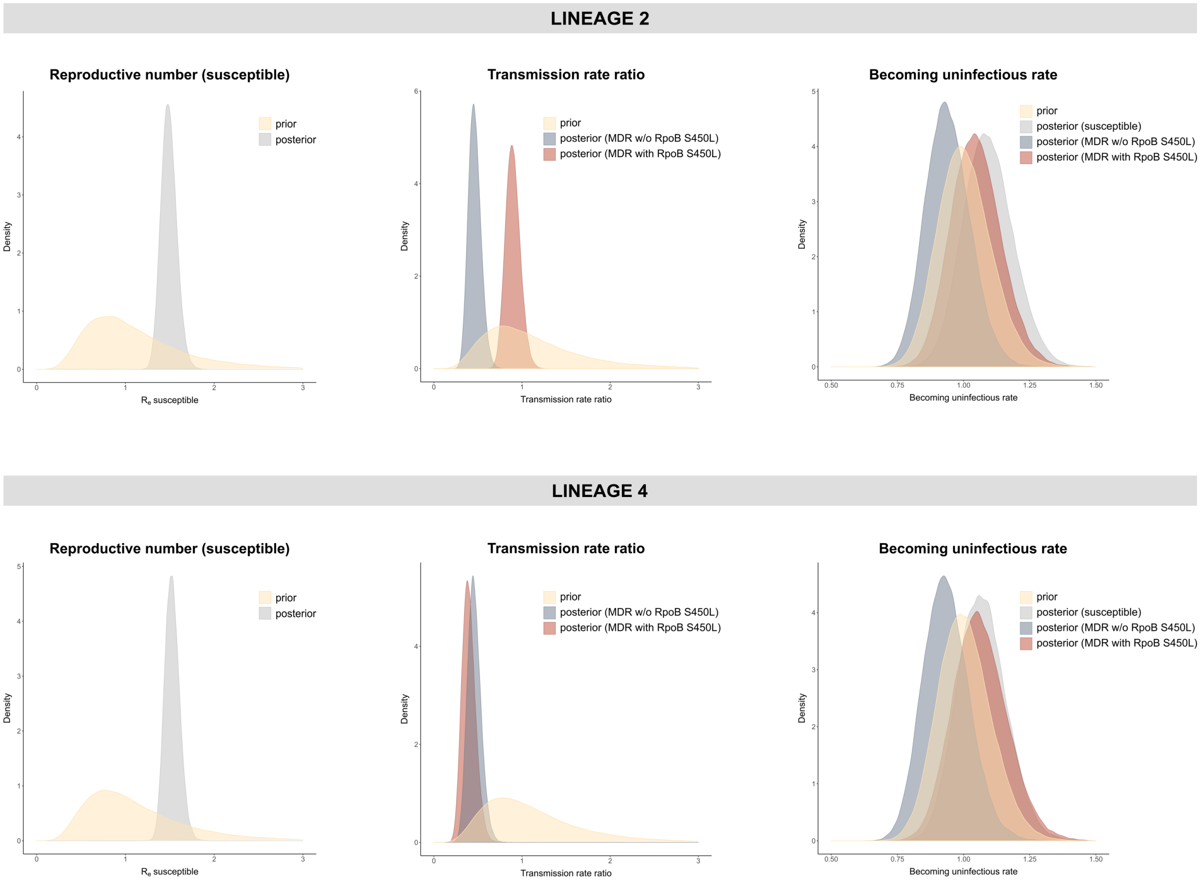

Fig. S10. Prior and posterior distributions corresponding to the phylodynamic analyses shown in Figure 4 of the main text.

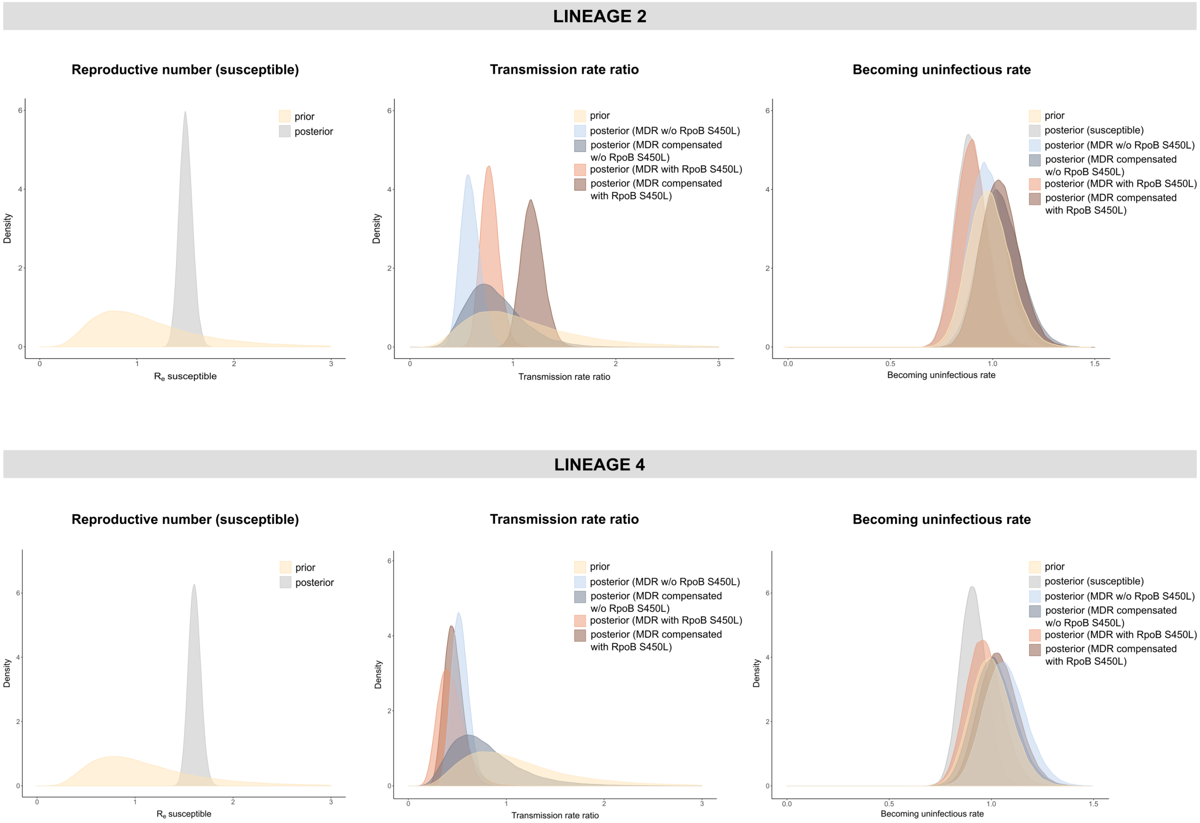

Fig. S11. Prior and posterior distributions corresponding to the phylodynamic analyses shown in Figure 5 of the main text.

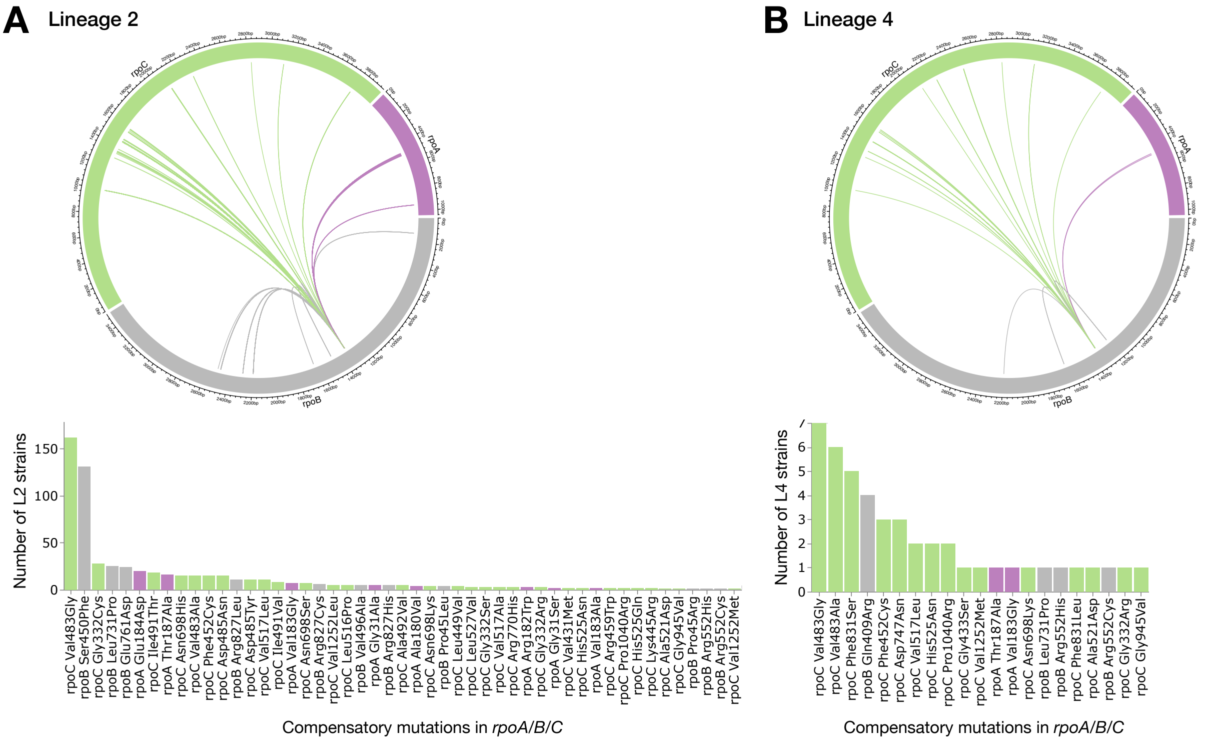

Fig. S12. Co-occurrence patterns of RpoB S450L with compensatory mutations in the lineage 2 (**A**) and lineage 4 background (**B**).

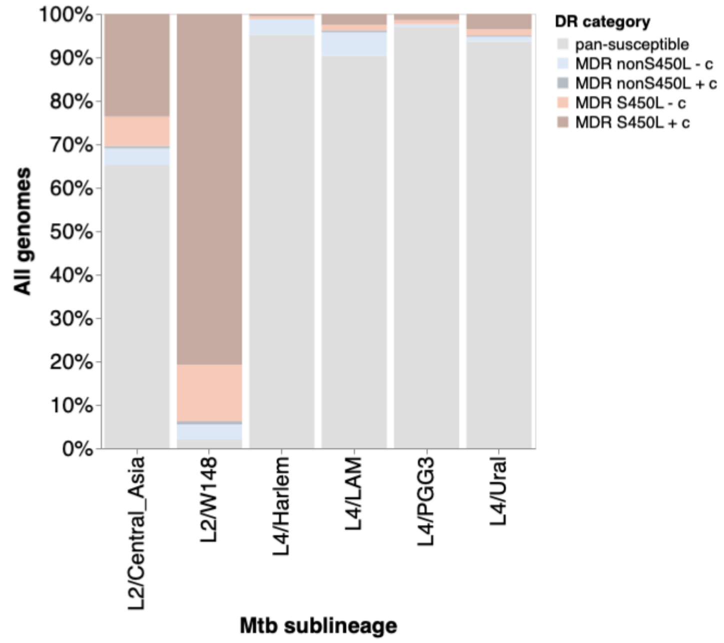

Fig. S13. Distribution of the different resistance categories in the main *M. tuberculosis* sublineages. CAO = Central Asia Outbreak; c = compensated strain.

2. Supplementary tables

Table S1. Prior distributions for the parameters of the multitype birth-death model.

**Parameter**	**Prior**
Reproductive number (drug-susceptible) $R_{e,S}$	Lognormal(0,0.5)
Transmission rate ratio λ_{MDR}/λ_S	Lognormal(0,0.5)
Becoming uninfectious rate δ	Lognormal(0,0.1)
Probability of removal upon sampling r	Uniform(0,1)
De novo mutation rate	Exponential(100)
Clock rate	Lognormal(-16,0.5)
Time of origin	Gamma(1,0.1)

Table S2. Prior distributions and values for the parameters used in TransPhylo.

Parameter	Prior or value
r parameter of secondary case distribution	Exponential(1)
p parameter of secondary case distribution	0.5
Shape parameter of generation time distribution	1.3
Scale parameter of generation time distribution	1.0
Shape parameter of sampling time distribution	1.3
Scale parameter of sampling time distribution	1.0
Sampling proportion	Uniform(0,1)
Within-host effective population size times generation time ($N_e g$)	Exponential(1)

Table S3. Multivariable logistic regression output showing host and bacterial factors associated with the probability of being a transmitter (derived with TransPhylo, with default parameters listed in Table S2). The variables work status, incarceration status, treatment outcome, and geographical region did not show a significant association. The significance of HIV status depends on the choice of parameters (see Tables S4-7).

Explanatory variable	Levels	No transmitter	Transmitter	Odds ratio	P-value
Resistance category L2	drug-susceptible	542	7	-	-
	MDR	625	22	2.72	0.20
Resistance category L4	drug-susceptible	1371	135	-	-
	MDR	100	2	0.20	0.048
Age	-	-	-	0.98	3.06×10^{-4}
Sex	Female	686	39	-	-
	Male	1952	127	1.41	0.036
HIV status	HIV-	2549	163	-	-
	HIV+	89	3	0.61	0.33

Table S4. Multivariable logistic regression output showing host and bacterial factors associated with the probability of being a transmitter (derived with TransPhylo, with a Gamma(2,1) distribution for generation and sampling times as sensitivity check). The variables work status, incarceration status, treatment outcome, and geographical region did not show a significant association. The significance of HIV status depends on the choice of parameters (see Tables S3,5-7).

Explanatory variable	Levels	No transmitter	Transmitter	Odds ratio	P-value
Resistance category L2	drug-susceptible	520	29	-	-
	MDR	596	51	1.55	0.36
Resistance category L4	drug-susceptible	1293	213	-	-
	MDR	97	5	0.31	0.029
Age	-	-	-	0.98	$3.20x10^{-8}$
Sex	Female	655	70	-	-
	Male	1851	228	1.40	0.0049
HIV status	HIV-	2418	294	-	-
	HIV+	88	4	0.41	0.057

Table S5. Multivariable logistic regression output showing host and bacterial factors associated with the probability of being a transmitter (derived with TransPhylo, with a Gamma(1.3,2) distribution for generation and sampling times as sensitivity check). The variables work status, incarceration status, treatment outcome, and geographical region did not show a significant association. The significance of HIV status depends on the choice of parameters (see Tables S3-4,6-7).

Explanatory variable	Levels	No transmitter	Transmitter	Odds ratio	P-value
Resistance category L2	drug-susceptible	540	9	-	-
	MDR	622	25	2.48	0.19
Resistance category L4	drug-susceptible	1355	151	-	-
	MDR	95	7	0.66	0.39
Age	-	-	-	0.98	2.85×10^{-5}
Sex	Female	683	42	-	-
	Male	1929	150	1.59	0.0035
HIV status	HIV-	2521	191	-	-
	HIV+	91	1	0.16	0.065

Table S6. Multivariable logistic regression output showing host and bacterial factors associated with the probability of being a transmitter (derived with TransPhylo, with posterior probability threshold of being a transmitter set to 75% as sensitivity check). The variables work status, incarceration status, treatment outcome, and geographical region did not show a significant association. The significance of HIV status depends on the choice of parameters (see Tables S3-5,7).

Explanatory variable	Levels	No transmitter	Transmitter	Odds ratio	P-value
Resistance category L2	drug-susceptible	516	33	-	-
	MDR	590	57	1.52	0.36
Resistance category L4	drug-susceptible	1264	242	-	-
	MDR	93	9	0.44	0.10
Age	-	-	-	0.98	1.07×10^{-10}
Sex	Female	646	79	-	-
	Male	1817	262	1.60	9.23×10^{-4}
HIV status	HIV-	2375	337	-	-
	HIV+	88	4	0.39	0.028

Table S7. Multivariable logistic regression output showing host and bacterial factors associated with the probability of being a transmitter (derived with TransPhylo, with posterior probability threshold of being a transmitter set to 50% as sensitivity check). The variables work status, incarceration status, treatment outcome, and geographical region did not show a significant association. The significance of HIV status depends on the choice of parameters (see Tables S3-6).

Explanatory variable	Levels	No transmitter	Transmitter	Odds ratio	P-value
Resistance category L2	drug-susceptible	458	91	-	-
	MDR	519	128	1.24	0.49
Resistance category L4	drug-susceptible	1094	412	-	-
	MDR	82	20	0.63	0.15
Age	-	-	-	0.98	$2.04x10^{12}$
Sex	Female	573	152	-	-
	Male	1580	499	1.39	$7.57x10^{-5}$
HIV status	HIV-	2075	637	-	-
	HIV+	78	14	0.61	0.033
