# Supplementary material for: The relative transmission fitness of multidrug-resistant Mycobacterium tuberculosis in a drug resistance hotspot
Source: Nat Commun. 2023 Apr 8;14:1988. doi: 10.1038/s41467-023-37719-y (PMC10082831; doi:10.1038/s41467-023-37719-y)
Supplement: Supplementary file 3 — Description of Additional Supplementary Files [file 41467_2023_37719_MOESM3_ESM.pdf]

Description of Additional Supplementary Files:

Supplementary Dataset 1: Metadata table, containing for each sample the genome accession number and associated bacterial and host-related factors.
