# Supplementary material for: The relative transmission fitness of multidrug-resistant Mycobacterium tuberculosis in a drug resistance hotspot
Source: Nat Commun. 2023 Apr 8;14:1988. doi: 10.1038/s41467-023-37719-y (PMC10082831; doi:10.1038/s41467-023-37719-y)
Supplement: Supplementary file 5 — Reporting Summary [file 41467_2023_37719_MOESM5_ESM.pdf]

nature portfolio

Corresponding author(s): Sebastien Gagneux
Etthel Windels

Last updated by author(s): Mar 14, 2023

Reporting Summary

Nature Portfolio wishes to improve the reproducibility of the work that we publish. This form provides structure for consistency and transparency in reporting. For further information on Nature Portfolio policies, see our Editorial Policies and the Editorial Policy Checklist.

Statistics

For all statistical analyses, confirm that the following items are present in the figure legend, table legend, main text, or Methods section.

n/a	Confirmed	
☐	☒	The exact sample size (*n*) for each experimental group/condition, given as a discrete number and unit of measurement
☐	☒	A statement on whether measurements were taken from distinct samples or whether the same sample was measured repeatedly
☐	☒	The statistical test(s) used AND whether they are one- or two-sided<br>*Only common tests should be described solely by name; describe more complex techniques in the Methods section.*
☐	☒	A description of all covariates tested
☐	☒	A description of any assumptions or corrections, such as tests of normality and adjustment for multiple comparisons
☐	☒	A full description of the statistical parameters including central tendency (e.g. means) or other basic estimates (e.g. regression coefficient) AND variation (e.g. standard deviation) or associated estimates of uncertainty (e.g. confidence intervals)
☐	☒	For null hypothesis testing, the test statistic (e.g. *F*, *t*, *r*) with confidence intervals, effect sizes, degrees of freedom and *P* value noted<br>*Give P values as exact values whenever suitable.*
☐	☒	For Bayesian analysis, information on the choice of priors and Markov chain Monte Carlo settings
☒	☐	For hierarchical and complex designs, identification of the appropriate level for tests and full reporting of outcomes
☒	☐	Estimates of effect sizes (e.g. Cohen's *d*, Pearson's *r*), indicating how they were calculated

Our web collection on statistics for biologists contains articles on many of the points above.

Software and code

Policy information about availability of computer code

Data collection	No software was used for data collection.
Data analysis	R v.3.6.2<br>Trimmomatic v33<br>SeqPrep v1.2<br>BWA v0.7.13<br>Pysam v0.90<br>Picard v1.135<br>GATK v3.4.0<br>Samtools v1.2<br>VarScan v2.4.1<br>SnpEff v4.1<br>RAxML v8.2.11<br>ggtree v3.3.1<br>circlize v.0.4.15<br>BEAST v2.6.6<br>bdmm v1.0<br>Tracer 1.7.1<br>TransPhylo v1.4.5

phylolm 2.6.2

The BEAST2 XML files and R code used for the phylodynamic analyses are available at https://github.com/EtthelWindels/mdr-tb_georgia_2014-2016.

For manuscripts utilizing custom algorithms or software that are central to the research but not yet described in published literature, software must be made available to editors and reviewers. We strongly encourage code deposition in a community repository (e.g. GitHub). See the Nature Portfolio guidelines for submitting code & software for further information.

Data

Policy information about availability of data

All manuscripts must include a data availability statement. This statement should provide the following information, where applicable:

- Accession codes, unique identifiers, or web links for publicly available datasets
- A description of any restrictions on data availability
- For clinical datasets or third party data, please ensure that the statement adheres to our policy

A total of 3,025 genome sequences were deposited to the European Nucleotide Archive (ENA). Newly sequenced MDR M. tuberculosis genomes (n=43) were registered under project accession number PRJEB39561 (https://www.ebi.ac.uk/ena/browser/view/PRJEB39561), a project ID already used in a previous study. All pan-susceptible M. tuberculosis genomes (n=2982) were registered under the project accession number PRJEB50582 (https://www.ebi.ac.uk/ena/browser/view/PRJEB50582). The individual BioSample accession IDs are provided in Supplementary Data 1.

Human research participants

Policy information about studies involving human research participants and Sex and Gender in Research.

Reporting on sex and gender	Patient sex was assigned by a clinician at the Georgian National Centre for Tuberculosis and Lung Diseases in Tbilisi. Patient sex was used as an explanatory variable and was adjusted for when analyzing different factors known to influence tuberculosis transmission.
Population characteristics	The following patient variables were recorded and controlled for: age, sex, HIV status, previous TB diagnosis, incarceration status, treatment outcome, work status, geographical region
Recruitment	No recruitment per se. All culture-positive TB cases reported to the Georgian National Centre for Tuberculosis and Lung Diseases in Tbilisi
Ethics oversight	The institutional Review Board of the National Centre for Tuberculosis and Lung Disease in Tbilisi, Georgia and the Ethics Commission of North- and Central Switzerland granted ethical approval for this study.

Note that full information on the approval of the study protocol must also be provided in the manuscript.

Field-specific reporting

Please select the one below that is the best fit for your research. If you are not sure, read the appropriate sections before making your selection.

☐ Life sciences ☐ Behavioural & social sciences ☒ Ecological, evolutionary & environmental sciences

For a reference copy of the document with all sections, see nature.com/documents/nr-reporting-summary-flat.pdf

Ecological, evolutionary & environmental sciences study design

All studies must disclose on these points even when the disclosure is negative.

Study description	To analyze the transmission of multidrug-resistant (MDR) Mycobacterium tuberculosis strains relative to their susceptible counterparts, we prospectively collected all (n = 4385) culture-positive TB cases reported to the Georgian National Centre for Tuberculosis and Lung Diseases (NCTLD) in Tbilisi from 2014 to 2016. These strains were subject to phenotypic drug-susceptibility testing. The MDR (n =1049) and pan-susceptible (n = 3242) strains were subject to whole-genome sequencing. The final dataset, comprised of 3962 high-quality genomes, was used to quantify the transmission of the strains using various phylodynamics methods.
Research sample	We conducted a nationwide population-based genomic epidemiological study in the country of Georgia. All TB patients that were culture-positive for Mycobacterium tuberculosis were included, irrespective of age and gender.
Sampling strategy	The sampling strategy was aimed at being as comprehensive and complete as possible. All patients that were culture-positive for TB were included. The final dataset is composed of 90% of all drug-sensitive culture-positive TB cases and 93% of all MDR culture-positive TB cases reported in Georgia (only excluding genomes that did not pass quality filtering).
Data collection	M. tuberculosis strains were collected at the NCTLD in Georgia where limited clinical data were also gathered through routine diagnostic work. DNA was sent to the Swiss Tropical and Public Health institute and sequenced with Illumina NovaSeq 6000 or HiSeq 2500 instruments at the Department of Biosystems Science and Engineering of the ETH Zurich, in Basel. The fastQ files were stored

	on sciCORE (https://scicore.unibas.ch).
Timing and spatial scale	Strains were collected between 01/01/2014 and 31/12/2016 and covered the entire country of Georgia.
Data exclusions	334 strains were excluded from the study for various reasons: low DNA concentration, failed sequencing library, low coverage, mixed infection, non MDR genotype, non susceptible genotype, contaminations.
Reproducibility	No experiments were performed in this study
Randomization	Patient isolates were classified as drug-resistant or drug-susceptible according to their phenotypic and genomic susceptibility profile. Analyses were corrected for age, sex and HIV status.
Blinding	Blinding was not relevant to this study because this was not an intervention study.

Did the study involve field work? ☐ Yes ☒ No

Reporting for specific materials, systems and methods

We require information from authors about some types of materials, experimental systems and methods used in many studies. Here, indicate whether each material, system or method listed is relevant to your study. If you are not sure if a list item applies to your research, read the appropriate section before selecting a response.

Materials & experimental systems

n/a	Involved in the study
☒	☐ Antibodies
☒	☐ Eukaryotic cell lines
☒	☐ Palaeontology and archaeology
☒	☐ Animals and other organisms
☒	☐ Clinical data
☒	☐ Dual use research of concern

Methods

n/a	Involved in the study
☒	☐ ChIP-seq
☒	☐ Flow cytometry
☒	☐ MRI-based neuroimaging
